# Camel milk in the horn of Africa: production systems, physicochemical, and nutritional quality, traditional utilization, safety, and market value chain dynamics

**DOI:** 10.1007/s44463-026-00077-6

**Published:** 2026-05-13

**Authors:** Shamsedin Mahdi Hassan, Yetenayet Bekele Tola, Sirawdink Fikreyesus Forsido, Joshua Arimi, Tilahun Abera Teka, Markos Makiso Urugo

**Affiliations:** 1 Department of Postharvest Management, College of Agriculture and Veterinary Medicine, Jimma University, P.O. Box 307 Jimma, Ethiopia; 2 Department of Human Nutrition, College of Dryland Agriculture, Jigjiga University, P.O. Box 1020 Jigjiga, Ethiopia; 3 Department of Food Science, Meru University of Science and Technology, Meru, Ethiopia; 4 Centre of Excellence in Camel Research, Meru University of Science and Technology, Meru, Kenya; 5 Department of Food Science and Applied Nutrition, College of Natural and Applied Sciences, Addis Ababa Science and Technology University, Addis Ababa, Ethiopia; 6 Food Science and Nutrition Research Directorate, Ethiopian Public Health Institute, P.O. Box 1242 Addis Ababa, Ethiopia; 7 Department of Food Science and Postharvest Technology, College of Agricultural Science, Wachemo University, P.O. Box 667 Hosaena, Ethiopia; 8 Somali Region Livestock and Agricultural Research Institute, Food Science and Nutrition Research Directorate, P.O. Box 398 Jigjiga, Ethiopia

**Keywords:** Camel milk production, Horn of Africa, Milk safety and quality, Traditional utilization, Value chain

## Abstract

Camel production plays a crucial role in supporting the livelihoods and food security of pastoral and agro-pastoral communities across Ethiopia, Kenya, Somalia, and Djibouti in the Horn of Africa. This review systematically synthesizes peer-reviewed studies and regional reports published between 2000 and 2026, identified through structured database searches and screened using defined inclusion criteria, to evaluate camel milk production systems, traditional utilization, physicochemical and nutritional quality, safety, and value chain dynamics across the region. Camel milk is widely consumed raw or fermented and serves as a key dietary resource. Traditional practices across the region involve spontaneous fermentation for product formation, the production of sour milk products and camel milk tea, and the incorporation of camel milk into various local dishes; however, systematic documentation of these practices remains limited. Compared with bovine and caprine milk, camel milk generally contains lower fat and lactose levels, higher vitamin C and mineral concentrations, and distinct protein characteristics, including lower κ-casein content and the absence of β-lactoglobulin, which influence digestibility and processing properties. Reported fat content ranges from approximately 2.5 to 4.5% and protein from 2.5 to 3.9%, while vitamin C levels substantially exceed those of bovine milk. These features confer nutritional advantages but also create technological challenges, such as weak coagulation and extended fermentation time. Despite increasing urban and cross-border demand, the sector remains constrained by feed shortages, limited veterinary services, inadequate processing facilities, informal marketing systems, and hygiene limitations that contribute to microbial contamination. Key gaps include a lack of harmonized quality standards, limited comparative data on milk composition and microbiological safety, weak cold-chain infrastructure, and poor value-chain coordination. Strengthened hygiene, standardized quality protocols, improved processing and cold-chain systems, and coordinated institutional support are essential to enhance commercialization, safety, and regional integration.

## Introduction

The Horn of Africa is one of the world’s major camel-rearing regions, encompassing Ethiopia, Somalia, Kenya, and Djibouti, where pastoralism remains the dominant livelihood strategy in arid and semi-arid drylands. Across this region, camels are uniquely adapted to harsh desert environments and serve as a backbone of socio-economic systems, contributing to food security, income generation, and resilience under climate variability (Padalino & Menchetti, 2024; Kena, 2022). Often referred to as the “White Gold of the desert,” camels thrive in environments unsuitable for other livestock species and are crucial for sustaining millions of people in drought-prone areas of the Horn of Africa (Bekele et al., 2021; Alemayehu et al., 2025).

Environmental and economic pressures increasingly challenge the sustainability of camel-based production systems throughout the Horn of Africa (Kena, 2022). Although Africa hosts over 80% of the global camel population, approximately 60% is concentrated in eastern African countries, including Sudan, Somalia, Ethiopia, and Kenya (Faye, 2015). However, official livestock statistics in several Horn of Africa countries may underestimate actual camel populations due to the mobility of pastoral herds, weak census systems, and cross-border movements. Ethiopia serves as a documented example of such underestimation (Kena, 2022; Getaneh et al., 2021), but similar structural limitations are reported across the region.

Within this broader regional context, Ethiopia hosts approximately 1.7–2.0 million camels, primarily concentrated in the Somali, Afar, and Oromia regions, where they support pastoral and agro-pastoral livelihoods (Begna et al., 2024; Rafu et al., 2021). Among livestock species in these drylands, camels provide milk, meat, transport, and income, forming a cornerstone of household food security systems (Bekele et al., 2021). However, livestock value chains across the region, including Ethiopia, face constraints such as inadequate infrastructure, irregular supply systems, and limited market-oriented production, which hinder their contribution to national and regional markets (Ahmed, 2019).

Projections indicate that the global camel population may reach 60 million within the next 25 years, underscoring the species’ growing relevance and climate adaptability (Masebo et al., 2023). In the Horn of Africa, increasing recognition of camels as climate-resilient livestock has renewed research interest in camel productivity, milk utilization, and value-chain development across multiple countries, not only in Ethiopia (Chekole et al., 2021). Their efficiency in harsh environments makes them central to sustainable milk and meat production under changing climatic conditions (Padalino & Menchetti, 2024). Nevertheless, information remains fragmented across production practices, utilization patterns, nutritional and safety issues, and market dynamics, and much of the available evidence is context-specific, highlighting significant knowledge gaps across the region.

This review, therefore, synthesizes evidence from Ethiopia, Kenya, Somalia, and Djibouti to provide a regionally balanced assessment of camel production systems, traditional utilization practices, physicochemical and nutritional characteristics, safety and quality concerns, and value-chain dynamics in the Horn of Africa. By consolidating dispersed evidence, the review identifies cross-cutting regional constraints and country-specific differences while highlighting priority areas for applied research, technological adaptation, and policy harmonization to strengthen camel milk commercialization and pastoral livelihoods.

## Methodology and literature search strategy

This study employed a structured narrative review design to systematically identify, screen, and synthesize published evidence on camel milk production systems, physicochemical and nutritional quality, traditional utilization, safety, and value-chain dynamics in the Horn of Africa. Although not conducted as a full systematic review or meta-analysis, the review followed transparent and replicable procedures to enhance methodological rigor.

A comprehensive search of peer-reviewed scientific literature and relevant regional and institutional reports was conducted. Literature published between 2000 and 2026 was considered, with priority given to studies published after 2010 to capture recent scientific, technological, and sectoral developments. Electronic databases searched included Scopus, Web of Science, ScienceDirect, and Google Scholar.

Search terms were developed to reflect the review’s thematic scope and applied in various Boolean combinations. Key terms included “camel milk,” “camel production systems,” “physicochemical composition,” “nutritional quality,” “traditional utilization,” “fermentation,” “milk safety,” “microbiological quality,” “value chain,” “market dynamics,” “pastoral systems,” and “Horn of Africa.” Country-specific search strings incorporating “Ethiopia,” “Kenya,” “Somalia,” and “Djibouti” were also applied to ensure balanced regional coverage.

The study selection process involved three stages: (1) initial screening of titles and abstracts to remove clearly irrelevant publications; (2) full-text assessment of potentially eligible studies; and (3) final selection based on predefined inclusion and exclusion criteria. The reference lists of all selected articles were also screened to identify relevant studies not captured during the initial database search.

The inclusion criteria comprised original research articles, review papers, theses, and institutional reports published in English that addressed camel milk production, composition, processing, safety, marketing, and value-chain organization in the Horn of Africa. Where appropriate, foundational and seminal studies were incorporated to provide essential theoretical and contextual background. Studies were excluded if they focused on camel products other than dairy products, were conference abstracts without accessible full texts, or lacked a clearly defined and adequately described methodology.

Data extraction focused on study location, production system characteristics, reported physicochemical and nutritional parameters, documented traditional utilization practices, microbiological findings, and value-chain constraints. Extracted information was organized thematically according to the objectives of the review.

The synthesis used a descriptive and comparative approach to identify patterns, similarities, and differences across countries in the Horn of Africa. Emphasis was placed on cross-cutting regional constraints, technological bottlenecks, safety concerns, policy gaps, and opportunities for value-chain upgrading. Quantitative findings were reported where available; however, due to heterogeneity in study design and measurement methods, formal meta-analysis was not performed.

### Camel production in the horn of Africa

The domestication of animals, driven by the need for a stable food supply and assistance in farming and transport, represents a pivotal milestone in human history (Endalew & Ayalew, 2016). This process enabled humans to utilize species for sustenance, agricultural labor, and transportation, shaping the development of societies worldwide (Endalew & Ayalew, 2016).

The Horn of Africa is one of the world’s major regions for camel production, encompassing Ethiopia, Somalia, Kenya, Sudan, and Djibouti, where camels constitute a strategic component of arid and semi-arid livestock systems. The regional camel population has increased over time, reflecting both herd expansion and growing reliance on climate-resilient livestock systems (Padalino & Menchetti, 2024; Kena, 2022). This growth partly reflects changing climatic conditions, as camel rearing offers a more resilient alternative to cattle production in the drylands of northern Sub-Saharan Africa (Rahimi et al., 2022; Kagunyu & Wanjohi, 2014). Africa hosts over 80% of the world’s camel population, with approximately 60% concentrated in eastern African countries, including Sudan, Somalia, Ethiopia, and Kenya (Faye, 2015). The arid conditions of Sub-Saharan drylands, characterized by limited rainfall and low suitability for crop production, favor livestock such as camels, which are well adapted to harsh environmental conditions (Kena, 2022).

In these regions, pastoralism has traditionally been the dominant livelihood strategy, relying heavily on camels due to their superior adaptation to arid and semi-arid environments (Marete et al., 2024). Camels can produce milk under extreme environmental conditions where the productivity of other livestock species declines significantly (Raziq et al., 2008; Faraz, 2020). Within the Horn of Africa, Kenya and Somalia are among the leading producers of camel milk, contributing substantially to regional supply and cross-border trade. Table 1 presents production volumes for these countries, with Kenya producing approximately 1.165 million litres and Somalia producing about 0.956 million litres of camel milk (Oselu, 2022a, 2022b).

**Table 1:** Recent camel milk production volumes in leading countries

Country	Annual production volume (million litre tonnes)	Reference
Kenya	1.165	Oselu (2022a, 2022b)
Somalia	0.958	Oselu (2022a, 2022b)
Ethiopia	0.176	FAO (2018)
Djibouti	0.0161	FAO-ONU (2013)
Eritrea	0.0098	Oselu (2022a, 2022b)

In 2017, global camel milk production was estimated at approximately 3 million tonnes, accounting for around 40% of total milk output in Somalia, 17% in Mali, and 12% in Ethiopia (Singh et al., 2017). Production increased to 4.11 million tonnes in 2022, with actual output potentially reaching 5.4 million tonnes due to underreporting. This increase represents a substantial expansion compared with production levels in the 1960s and reflects an average annual growth rate of approximately 2.45% (Faye, 2008; FAO, FAOSTAT, 2022).

In Ethiopia, camels are primarily reared in the Afar, Somali, and eastern and southern Oromia regions by pastoral and agro-pastoral groups such as the Beja, Rashaida, Afar, Somali, Karayu, and Borana communities (Kena, 2022). These populations form part of the broader Horn of Africa camel production system and contribute to regional milk supply and livestock-based livelihoods (Kena, 2022). In the country’s arid and semi-arid rangelands, where pastoralism remains the primary livelihood, camels have evolved as one of the most ecologically suited livestock species, characterized by their ability to utilize spatially and temporally scattered pasture and water resources. This ecological context supports mobile and nomadic production systems.

Camels are highly adapted to these rangelands and support pastoral mobility, and dromedaries yield relatively higher milk production compared with other livestock species under similar environmental conditions (Mirkena et al., 2018; Gebremichael et al., 2019). Camel milk is typically consumed raw in most pastoral societies (Sisay and Awoke, 2015) and serves as a vital source of nutrition, year-round sustenance, and economic stability for pastoralist communities in eastern Africa (Farah et al., 2007; Elhadi et al., 2015; Kebede et al., 2015; Wako, 2015). Beyond its nutritional and economic importance, camel milk also holds cultural value and, with adequate investment in infrastructure, animal health services, and market development, has strong potential to strengthen sustainable pastoral livelihoods and expand regional commercialization.

### Physicochemical and nutritional composition of camel milk

The nutritional and physicochemical analyses show that camel milk has slightly lower lactose levels and much lower fat levels than bovine and caprine milk, but comparable levels of protein and total solids (Abduku & Eshetu, 2024; Al Haj & Al Kanhal, 2010). Its most distinctive feature is its exceptionally high vitamin C concentration, far surpassing the very low levels found in bovine and caprine milk, highlighting its strong nutritional advantage (Wang et al., 2011; Ahmed et al., 2017). Camel milk has lower vitamin A content than cow milk but is rich in minerals such as calcium, phosphorus, potassium, magnesium, and sodium at levels comparable to or near those set for other milk types (Abduku & Eshetu, 2024; Al Haj & Al Kanhal, 2010; Asresie & Adugna, 2014). Table 2 indicates that camel milk has a lower fat content, is enriched with essential minerals, and has uniquely high vitamin C content, making it a valuable nutritional resource, especially in regions where it forms a significant part of the diet.

**Table 2:** Comparison of the physicochemical and nutritional composition of camel, bovine, and caprine milk

Parameter	Camel milk	Bovine milk	Caprine milk	References
Total solids (%)	12-14.89	11.14–12.70	12.30–14	Alhaj et al. (2013), Fox et al. (2016), Mohamed and El Zubeir (2020), Liu et al. (2023), Akshit et al. (2024), Al-Fayad and Abdulwahid (2022)
Fat (%)	3.1–6.4	3.8–8.60	4.1–8.50	Fantuz et al. (2016), Mohamed and El Zubeir (2020), Berhe et al. (2017), Legesse et al. (2017)
Protein (%)	3.5–3.67	3.35–3.4	3.3–3.53	Akshit et al. (2024), Antunes et al. (2022), Khaliq et al. (2024), Fantuz et al. (2016), Mohamed and El Zubeir (2020)
Lactose (g/100 g)	4.4–5.01	4.8–4.9	3.78–4.5	Fantuz et al. (2016), Mohamed and El Zubeir (2020), Swelum et al. (2021), Kapadiya et al. (2016)
Vitamin C (mg/100 g)	3.0-34.5	0.94-2	1.29–1.3	Alhassani (2024), Barlowska et al. (2011), Benmeziane–Derradji (2021), Flis and Molik (2021)
Vitamin A (µg/100 g)	26.7–360	126	185	Alhaj et al. (2022), Barlowska et al. (2011), Vincenzetti et al. (2022)
Calcium (mg/100 g)	112.93–160	122–140	132–134	Abdelazez et al. (2024), Alhaj et al. (2022), Barlowska et al. (2011), Konuspayeva et al. (2022), Vincenzetti et al. (2022)
Phosphorus (mg/100 g)	81.2–87.4	90–119	97.7–121	Barlowska et al. (2011), Nayik et al. (2022), Rakhmatulina et al. (2024)
Potassium (mg/100 g)	116.13–152	136–140	152–181	Alhaj et al. (2022), Barlowska et al. (2011), Yu et al. (2024)
Magnesium (mg/100 g)	9.65–12.3	18–21	15.8–16	Alhaj et al. (2022), Barlowska et al. (2011), Yu et al. (2024)
Sodium (mg/100 g)	53.10–59	58	41-59.4	Alhaj et al. (2022), Barlowska et al. (2011), Yu et al. (2024)

### Traditional and emerging utilization of camel milk and its products

Camels are essential dairy animals in desert and arid regions of the Horn of Africa, where they often produce more milk under harsh environmental conditions than other livestock species. Their milk possesses unique physicochemical and nutritional properties that distinguish it from bovine and caprine milk and enhance its suitability for pastoral production systems (Seifu, 2022). Across Ethiopia, Somalia, Kenya, Sudan, and Djibouti, camel milk plays a critical role in sustaining pastoral and agro-pastoral livelihoods in environments characterized by climatic variability and resource scarcity (Tura et al., 2010).

These animals exhibit remarkable tolerance to extreme conditions, demonstrating resilience and efficient energy utilization, which makes them highly adapted to challenging environments (Madalcho et al., 2019). Within pastoral communities, breeding selection has traditionally focused on desirable traits such as physical strength, temperament, coat characteristics, milk yield potential, and hardiness, particularly in regions such as Somalia, where herders actively select superior breeding males (Khaskheli, 2020). Despite their importance, dromedaries have lower reproductive efficiency compared with other domesticated species. Factors such as a restricted mating season, delayed sexual maturity, and a prolonged gestation period of approximately 13 months contribute to relatively slow herd expansion under traditional management systems (Ahmed, 2018).

#### Traditional utilization

Camel milk remains a vital dietary resource in arid and semi-arid regions, particularly among nomadic and semi-nomadic populations across the Horn of Africa, where it is traditionally consumed raw or after spontaneous fermentation. Common traditional uses include fresh raw milk, naturally fermented and sour milk products, and camel tea, which often serves as a base for soups and stews. However, despite its widespread traditional use, transforming camel milk into processed dairy products remains a significant technological challenge. Fermented camel milk has long been valued for its nutritional contribution and its perceived therapeutic properties. Its natural antimicrobial components, including lactoferrin and immunoglobulins, contribute to extended shelf life compared with bovine milk and support traditional preservation practices (Arain et al., 2023; Hamed et al., 2024; Muthukumaran et al., 2022). Products such as butter and cheese often exhibit lower yields and different textural properties compared with bovine milk products due to compositional differences, including lower κ-casein content and weaker coagulation capacity (Seifu, 2023).

#### Nutritional significance and growing demand

In recent years, demand for camel milk has increased, particularly in urban markets and regions with limited local production (Mbogo et al., 2012). Nutritionally, camel milk provides higher concentrations of vitamin C and niacin than milk from other livestock species, supporting populations with limited access to fresh fruits and vegetables (Breulmann & Böer, 2010). Its relatively higher water content during dry seasons contributes to hydration and physiological balance. Compared with bovine and caprine milk, camel milk contains lower fat, protein, and total solids, enhanced digestibility, and reduced allergenic potential, with compositional similarities to human milk (Roy et al., 2020). These characteristics strengthen its positioning as both a traditional staple and a functional food in emerging markets.

#### Processing innovations and commercialization

Over the past decades, camel milk processing has diversified in response to growing regional and global demand. Technological advances have enabled the production of pasteurized milk, milk powder, yoghurt, cheese, ice cream, chocolate, and probiotic beverages, each adapted to its unique physicochemical properties.

Unlike bovine milk, camel milk lacks β-lactoglobulin, contains low levels of κ-casein, and has larger casein micelles. These characteristics complicate coagulation and cheese-making. Nevertheless, optimization of processing techniques, including controlled fermentation, enzymatic modification, pasteurization adjustments, and spray drying, has improved product quality and expanded product diversification (Arain et al., 2023, 2024; Konuspayeva & Faye, 2021; Seifu, 2023).

Camel milk-based probiotic products represent a growing niche in the functional food market. These products capitalize on bioactive components and beneficial lactic acid bacteria, demonstrating reported antidiabetic, antimicrobial, anticancer, and immune-modulating properties (Ansari et al., 2024; Arain et al., 2022). Ongoing research focuses on standardizing processing protocols to preserve sensory attributes while maximizing health benefits.

To meet increasing demand, several large-scale production and processing enterprises have emerged globally and within Africa. Notable examples include Tiviski in Mauritania (Gaye, 2007), Al-Watania in Saudi Arabia, Tedjane in Algeria, Camelicious in Dubai (Konuspayeva & Faye, 2021), and Addis Kidan Milk Processing Enterprises in Ethiopia. These enterprises primarily produce pasteurized and packaged camel milk for urban consumers and export markets (Ipsen, 2017). Table 3 Summarizes traditional and processed camel milk products across Horn of Africa countries.

**Table 3:** Traditional and processed camel milk products across horn of Africa countries

Country	Documented traditional products	Documented processed/value-added products	Level of scientific documentation	References
Ethiopia	Raw camel milk; **dhanaan** (spontaneously fermented milk); traditional fermented milk; camel milk tea	Experimental yoghurt production; laboratory-scale fermented products; limited pasteurised milk marketed locally; cheese production largely experimental due to coagulation limitations	Moderate documentation for traditional products; limited but emerging evidence for processing	Karssa et al. (2024, 2025), Medalcho (2026), Seifu (2023), Faccia e al., (2020)
Kenya	Raw milk; **suusac** (traditional spontaneously fermented camel milk); fermented milk used in pastoral diets	Pasteurised camel milk for urban markets; small-scale yoghurt production; limited commercial processing enterprises	Strong documentation for traditional fermented products; moderate documentation for pasteurised milk commercialization	Elhadi et al. (2015), Lore et al. (2005), Maitha et al. (2019), Mwangi et al. (2016), Muloi et al. (2018), Oselu et al. (2022a, b)
Somalia	Raw camel milk; **garris; dhanaan** (traditional fermented milk); fermented milk consumed fresh or mixed with cereals	Limited documented industrial processing; emerging packaged products in urban markets, but weak peer-reviewed coverage	Strong evidence for traditional products; weak scientific documentation for industrial processing	Farah et al. (2007), Khaliq et al. (2024), Konuspayeva and Faye (2021), Shori (2012)
Sudan	Raw milk; **shubat**-type fermented camel milk products are documented in pastoral communities	Minimal documented industrial processing; experimental fermentation studies	Limited documentation for processed products; moderate documentation for fermented milk traditions	Hassan and Elzubar (2006), Osman et al. (2024), Shori (2012), Omar (2018)
Djibouti	Raw camel milk; traditional fermented consumption practices	Very limited evidence of structured processing or commercial product diversification	Weak scientific documentation overall; data gaps identified	Seifu (2023), Mohamed et al. (2017)

### Safety and quality aspects of camel milk

Many assume that the natural antimicrobial properties of camel milk may inhibit spoilage at ambient temperatures; however, these effects are limited, and inadequate hygiene during collection can lead to increased microbial contamination (Sela et al., 2003). Although several studies have examined camel milk microbiology, much of the available evidence comes from localized investigations, and variations in season, geographic location, herd management, and farm hygiene practices within the Horn of Africa significantly influence contamination levels.

Studies conducted in Ethiopia and neighboring countries demonstrate marked variability in microbial quality depending on production environment and handling practices. For example, research in the Somali Regional State of Ethiopia reported total bacterial counts exceeding acceptable limits, indicating contamination along the value chain and insufficient hygiene during milking and transportation (Abera et al. 2016a, b; Geresu et al. 2021). Similarly, investigations in pastoral areas of Afar and other lowland regions documented the presence of *Staphylococcus aureus*, *Escherichia coli*, and other indicator organisms in raw camel milk, with higher contamination levels associated with poor udder hygiene and traditional storage conditions (Adugna et al., 2013; Ayuob et al., 2020). The thermal death time of *Escherichia coli* in camel milk is comparable to that in cow milk, and *E. coli* is frequently detected in raw samples when hygienic practices are inadequate (Al-Rasheedi et al., 2015). Pathogenic microorganisms can enter milk during milking, storage, and processing, contributing to microbial deterioration (Mihajlović et al., 2022; Geresu et al., 2021). Pathogens may originate from the udder, particularly in cases of mastitis, with *Staphylococcus* and *Streptococcus* species being among the most commonly isolated pathogens. Other organisms such as *Corynebacterium*, *Klebsiella*, *Listeria*, *Pseudomonas*, *Salmonella*, and *Serratia* have also been reported, highlighting microbiological risks across production, transportation, and storage systems.

Evidence from multiple Horn of Africa studies further demonstrates regional differences in microbial loads (Table 4). Seasonal variation plays a major role, with higher bacterial counts often recorded during the wet season due to increased environmental contamination and handling frequency. Location-specific differences have also been observed between peri-urban milk collection centers and remote pastoral settings, where infrastructure and cold-chain facilities differ substantially. Such variability underscores that microbial quality is strongly influenced by farm management practices, environmental conditions, and marketing systems across the region (Abera et al. 2016a, b; Adugna et al. 2013).

**Table 4:** Evidence from multiple studies in the horn of Africa showing regional variation in microbial loads and dominant bacterial contaminants in raw camel milk

Country	Study location	Season/context	Microbial parameters assessed	Key findings	Main Reported pathogens/indicators	Reference
Ethiopia	Somali Regional State (Fafan Zone)	Mixed seasons; value-chain sampling (udder, milking bucket, market) of raw camel milk	- Total bacterial count (TBC) - Coliform count (CC) - Isolation and identification of bacterial species	− 85.7% of raw camel milk samples were contaminated above acceptable levels - Mean TBC ≈ 4.75 ± 0.17 log CFU/ml and mean CC ≈ 4.03 ± 0.26 log CFU/ml - TBC increased from udder to market level and was higher in Gursum than Babile (*P* < 0.05) − 38.9% of TBC and 88.2% of CC were in the range considered unsafe for human use - Indicates poor hygiene and contamination along production to market	*- Staphylococcus spp. *(89.8%) *- Streptococcus spp. *(53.7%) *- Escherichia coli *(31.5%) *- Salmonella spp. *(17.6%) *- Klebsiella spp. *(5.6%) *- Enterobacter spp. *(5.6%)	Abera et al. (2016a, b)
Ethiopia	Bule Hora and Dugda Dawa districts, Southern Ethiopia	August 2017 to February 2018, pastoral context	- Mastitis prevalence (CMT and clinical/subclinical) - Udder and milk abnormalities - Isolation and identification of bacteria from CMT-positive samples - Antibiotic resistance profiles	- Camel-level mastitis prevalence was 26.3% at animal level and 14.2% at udder half level - Clinical mastitis in camels was 9.8%, subclinical 19.6% - Udder abnormality and mastitis were significantly higher in late lactation than early lactation - Mastitis prevalence tended to increase with parity in camels - Isolates from camels showed high resistance to common antibiotics; concern for multidrug resistance	*-* Coagulase-negative *Staphylococcus spp. (most frequent)* *- Staphylococcus aureus* *- Staphylococcus hyicus* *- Staphylococcus intermedius* *- Escherichia coli* *-* Antibiotic resistance indicators (e.g., multidrug resistance, high resistance to penicillin, spectinomycin)	Balemi et al. (2021)
Ethiopia	Jigjiga City, Somali Region	Cross-sectional study on dairy camels (subclinical mastitis)	- Prevalence of subclinical mastitis (CMT) - Bacterial isolation - Antibiotic susceptibility	Overall subclinical mastitis prevalence 10.6%; age and hygiene significant risk factors; many isolates showed antibiotic sensitivity variability	*- Staphylococcus aureus *(34.5%) *- Streptococcus agalactiae *(29.8%) *- Streptococcus dysgalactiae *(19.4%) *- Pasteurella multocida *(16.2%)	Jama et al. (2024)
Kenya	Across the camel milk market chain (production to final market)	Value-chain sampling	- Total bacterial count (TBC/TVC) - Enterobacteriaceae/coliform counts (EBC) - *Staphylococcus* spp. (PSC) - Yeast and molds	- TBC increased along the milk chain from milking to final market, with final market milk often exceeding acceptable limits (> 10⁶ cfu/ml), indicating poor quality and hygiene issues	*- Total bacterial counts (TBC) increased along chain* *- Enterobacteriaceae/*coliforms above grade II quality limits in many samples - Staphylococcus spp. detected (used as an indicator)	Kaindi et al. (2011)
Kenya	Northern & Eastern pastoral areas (Isiolo County and along value chain to Nairobi)	Informal market chains; milk held at ambient temperatures before sale	- Total viable bacterial count (TVBC/TBC) - Total coliform count (CC)	- Microbial counts significantly increased along the value chain from production to final market, especially under uncooled storage and handling at ambient temperatures (often 28–32.5 °C) - Higher bacterial loads were associated with longer holding times and poor handling practices (no cooling, use of non-food grade plastic containers) - Coliform counts and total bacterial counts escalated along chain stages, indicating declining quality	- Total bacterial load (TBC/TVBC) increased through the chain - Coliform bacteria (indicator of hygiene failure) - Staphylococcus spp. and other environmental bacteria cited in related literature as common contaminants	Nato et al. (2018)
Somalia	Garowe District, Puntland	Cross-sectional camel mastitis study (May–Aug 2023)	- Mastitis prevalence (CMT & clinical exam) - Bacteriological culture	Overall mastitis prevalence 39.7% (animal level); higher quarter-level positivity; significant association with tick infestation and hygiene	- *Staphylococcus* spp. (45.7%) - *Streptococcus agalactiae* (25.7%) - *Escherichia coli* (14.3%) - *Klebsiella* spp. (8.6%) - *Micrococcus* (5.7%)	Farah et al. (2023)
Somalia	Benadir Region	Cross-sectional camel mastitis study (May–Sep 2022)	- Mastitis prevalence (CMT) - Risk factor analysis	Camel-level mastitis prevalence 34.4%; clinical ~ 5.2% and subclinical ~ 29.2%; quarter-level prevalence ~ 46.3%; significant risk factors included lactation stage, parity, age and poor hygiene	Not specified by isolate genus	Mohamed et al. (2024)
Sudan	Khartoum State, Sudan	Summer 2017 & Winter 2018; informal raw camel milk production	- Total coliforms - *Escherichia coli* (subset of coliforms) - Staphylococcus spp. - Yeast and molds	Raw camel milk showed coliform bacteria and Staphylococcus spp. as dominant microbial contaminants. Salmonella was not detected in both seasons. Seasonal variation noted with coliforms more prevalent in summer	*- Coliform bacteria (total and E. coli)* *- Staphylococcus spp.* *- Streptococcus spp.* *- Yeasts and molds detected*	Elrofaei et al. (2021)
Djibouti	Six districts across Djibouti	Cross-sectional raw milk quality study (October–November 2015)	- Aerobic plate count - (Implied) overall microbial quality	Raw camel milk from farmers in Djibouti showed variable microbial loads; study highlighted that milk is commonly consumed without pasteurization, leading to elevated public health risk. Quantitative data show elevated aerobic bacterial counts (for camel milk among other milks)	- Aerobic bacterial counts (viable bacteria) - Indicator for poor hygienic quality in raw milk (camel included)	Mohamed et al. (2017)

Despite its nutritional benefits, the microbiological safety of camel milk remains a concern throughout production, transportation, and storage. Studies from Ethiopia illustrate that contamination levels frequently exceed recommended standards in informal market chains, particularly where milk is sold raw without cooling. These findings demonstrate that microbial quality is closely linked to handling practices and local production conditions. With rising demand for camel milk and expansion of commercial processing, maintaining milk safety is essential for domestic and regional markets (Nagy et al., 2022; Chaudhary et al., 2017).

### Consumer preferences and market potential for camel milk and milk products

Consumer preference for camel milk is increasing across the Horn of Africa, driven by recognition of its nutritional value, perceived medicinal properties, and growing urban demand. While rural pastoral and agro-pastoral communities primarily consume camel milk in traditional forms, urban consumers increasingly demand processed, packaged, and hygienically handled products (Arain et al., 2023). These consumption patterns vary across countries depending on production systems, infrastructure development, and market accessibility.

In pastoral and agropastoral communities, camel milk is primarily consumed fresh or fermented, whereas in urban centers, there is growing demand for pasteurized and packaged products. Traditional product forms include raw milk, spontaneously fermented milk, sour milk preparations, and camel milk tea, which remain dominant in Ethiopia, Somalia, Kenya, and parts of Sudan and Djibouti (Table 5). Producers can process camel milk into a wide range of value-added products, including milk powder, cheese, yogurt, ice cream, and chocolate. These product forms are gradually entering formal markets, particularly in Ethiopia, Somalia, and Kenya, where commercialization initiatives and peri-urban processing enterprises are expanding.

**Table 5:** Camel milk product types, consumption patterns, and market potential across horn of Africa countries

Country	Main product forms	Consumption pattern	Level of processing/commercialization	Market potential & key constraints	Key Scientific Sources
Ethiopia	Raw milk; fermented milk (dhanaan); camel milk tea; pasteurized milk; yoghurt (limited); experimental cheese and powder	Predominantly raw/fermented in pastoral areas; growing demand for pasteurized products in urban centers	Emerging small- and medium-scale processing; limited industrial capacity	High potential due to large herd population and urban demand; constrained by weak cold chain, quality control, and infrastructure	Berhe et al. (2017), Seifu (2023), Abera et al. (2016a, b), Karssa et al. (2025)
Kenya	Raw milk; suusac (traditional fermented milk); pasteurized packaged milk; yoghurt	Strong traditional fermented consumption; increasing urban demand for packaged milk	More developed commercialization compared to neighboring countries; peri-urban dairies operating	High regional trade potential; better market integration; constrained by seasonal supply fluctuations and quality standard enforcement	Lore et al. (2005), Mbogo et al. (2012,; Mwangi et al. (2016), Muloi et al. (2018), Noor et al. (2013), Oselu et al. (2022a, b)
Somalia	Raw milk; garris (fermented milk); traditional dairy-based preparations	Dominantly raw and fermented consumption; limited packaged products in urban markets	Very limited formal processing infrastructure	Strong cross-border trade potential; market expansion limited by instability, weak regulation, and infrastructure gaps	Ait El Alia et al. (2025), Farah et al. (2007), Younan and Mwangi (2012)
Sudan	Raw milk; shubat-type fermented milk	Primarily traditional household consumption	Minimal documented industrial processing	Untapped potential for formal processing; constraints include limited investment and weak cold chain systems	Osman et al. (2024), Seifu (2023)
Djibouti	Raw milk; fermented milk	Small-scale traditional consumption	Limited evidence of structured processing	Small market size but potential for niche urban markets; major data gaps in commercialization studies	Mohamed et al., (2017), Hamed et al. (2024)

Camel milk is highly versatile, and processors can convert it into dairy and non-dairy products tailored to different market segments. However, microbial contamination and inconsistent quality management continue to constrain market expansion. For example, studies from the Fafan zone of Ethiopia demonstrated that total bacterial counts vary along the market chain, emphasizing the need for improved hygiene, cooling systems, and standardized handling practices to strengthen consumer confidence and food safety (Abera et al. 2016a, b). Quality and safety concerns directly influence market integration, particularly in formal and export-oriented value chains.

Efforts by regional institutions and organizations such as the FAO aim to promote commercialization and value addition to improve pastoral livelihoods. Cross-border trade among Ethiopia, Somalia, and Kenya further supports regional market integration and demonstrates the growing economic relevance of camel milk across the Horn of Africa. Despite these advancements, the camel milk industry faces significant processing and distribution challenges, often struggling to meet stringent quality standards required for wider market penetration (Alia et al., 2025; Seifu, 2023). Only a limited proportion of total production is currently processed, indicating substantial untapped potential for product diversification and industrial development. Adaptation of processing technologies to camel milk’s unique physicochemical properties remains necessary to optimize product quality and scalability.

Camel milk differs from bovine milk in several key aspects, including higher mineral and vitamin C content, a distinct whey-to-casein ratio, and the presence of bioactive components that enhance its functional value (Zou et al., 2022). These characteristics contribute to its digestibility and appeal among health-conscious consumers but also create technological challenges. Its compositional features include lower saturated fatty acids, smaller fat globules, higher β-casein levels, and lower κ-casein content, which improve nutritional quality but reduce rennetability and coagulation efficiency. Processing limitations such as weak curd formation and extended fermentation time necessitate innovative technological solutions to achieve desirable texture and product stability (Seifu, 2023).

### Camel milk value chain from pastoralist production to commercial markets

The camel milk value chain in the Horn of Africa can be understood through sequential stages: production, collection and aggregation, processing, and commercialization. Pastoral production systems, infrastructural capacity, and mechanisms of market integration shape each stage.

At the production stage, the value chain is largely dominated by pastoralist and agro-pastoral systems. In Ethiopia and across the region, camel milk is primarily produced under small-scale traditional management practices characterized by low external inputs, limited mechanization, and mobility-based herd management. Production conditions are strongly influenced by environmental variability and resource availability, which shape milk yield, quality, and supply consistency. Herd mobility in search of pasture and water directly affects downstream activities such as collection, processing, and marketing (Gebremichael et al., 2019).

**Fig. 1:**
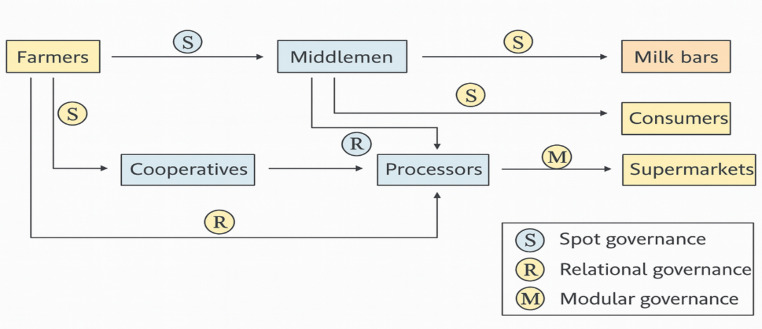
Camel milk value chain, illustrating governance structures from farmers to consumer markets in the horn of Africa

At the collection and aggregation stage, informal market channels predominate across most Horn of Africa countries. Milk is often transported without standardized cooling systems or formal quality control mechanisms. These constraints reduce shelf life, increase microbial risks, and limit consistent supply to structured markets. Emerging peri-urban camel dairies are beginning to bridge pastoral production systems with urban markets by aggregating milk, improving hygiene practices, and supplying growing urban demand (Agriconsortium, 2003; Isako & Kimindu, 2019). This transition reflects a gradual shift in the market toward semi-intensive systems, driven by urbanization, population growth, and expanding consumer demand (Muloi et al., 2018). The governance structure and functional relationships across these stages in the Horn of Africa are illustrated in Fig. 1.

At the processing and commercialization stage, modernization efforts remain uneven across the region. Some local enterprises are expanding pasteurization, packaging, cold-chain logistics, and product diversification; however, value addition remains limited relative to production potential. Countries with more advanced dairy industries, such as Kenya, Kazakhstan, Saudi Arabia, and the United Arab Emirates, demonstrate how investment in mechanization, regulatory frameworks, and cold-chain infrastructure can strengthen value-chain coordination and product standardization (Oselu et al. 2022a, b; Abdullah and Sheikh 2024; DairyGlobal.net, 2024). These cases are presented as comparative benchmarks that illustrate possible development pathways rather than direct models for replication in the Horn of Africa. In contrast, herd mobility, weak infrastructure, fragmented market access, and limited processing capacity continue to constrain commercialization and formal market integration within the region.

Climate resilience is a cross-cutting factor that influences value-chain sustainability. The expansion of camel production in the Horn of Africa is largely an adaptive response to recurrent droughts and climate variability. Camels provide relatively stable milk output under arid conditions compared with other livestock species, thereby supporting household food security and supply stability during environmental shocks (Hülsebusch et al., 2002; Isako & Kimindu, 2019). This resilience enhances production reliability, strengthens raw material availability for processors, and contributes to long-term value-chain sustainability. Moreover, the multipurpose nature of camels and increasing recognition of camel milk in domestic and international niche markets position the sector as both a climate-adaptive livelihood strategy and an emerging commercial commodity (Geresu et al., 2021). Integrating climate-resilient production systems with improved aggregation, quality control, and processing infrastructure is therefore essential for upgrading the camel milk value chain and achieving deeper regional market integration.

### Challenges and constraints in camel production and milk marketing

Across the Horn of Africa, including Ethiopia, Somalia, Kenya, Sudan, and Djibouti, camel production remains ecologically significant yet institutionally under-supported. Despite its adaptability to arid environments and its economic importance for pastoral livelihoods, the camel sector has historically received limited research investment, policy prioritization, and institutional coordination across the region (Yusuf & Tadesse, 2004; Kena, 2022). Similar patterns of marginalization appear to exist in neighboring countries, where livestock development programs tends to prioritize cattle and small ruminants over camels, potentially resulting in gaps in scientific knowledge, breed improvement programs, and structured support systems.

This institutional gap is not country-specific but region-wide and has contributed to weak policy harmonization, limited data systems, and fragmented development strategies for camel milk production and marketing across Horn of Africa countries. Many countries lack national standards for processed camel milk products (Seifu, 2023), hindering trade and export expansion within and beyond the region. Regulatory harmonization among Ethiopia, Somalia, Kenya, Sudan, and Djibouti remains limited, constraining cross-border trade despite strong informal market flows. Although global demand for camel milk is increasing due to its perceived health benefits, access to international markets remains restricted by the absence of standardized quality frameworks and harmonized import regulations. This regulatory fragmentation represents a structural barrier to regional market integration and global competitiveness.

Among the key production-related challenges across the region are widespread camel diseases, inadequate veterinary service coverage, and limited technical capacity in herd health management. In pastoral areas of northern Kenya and Somalia, similar constraints have been reported, including limited access to veterinary support and recurrent disease outbreaks, which reduce productivity and milk yield. In Ethiopia’s Afar region, natural pastures and browse species constitute the main feed resources. However, rangeland degradation due to deforestation, charcoal production, land-use change, and recurrent droughts has reduced feed availability. Comparable trends in feed scarcity and rangeland degradation are also documented in arid zones of Somalia, northern Kenya, Sudan, and parts of Djibouti, where climate variability intensifies production risks and reduces milk output.

Feed limitations and animal health constraints reduce production efficiency throughout the value chain. Studies from Ethiopia’s Somali region demonstrate that disease prevalence and feed shortages significantly affect milk yield (Kebede et al., 2015), and similar structural vulnerabilities have been reported across neighboring countries with limited pasture management systems and weak extension services. Marketing challenges further constrain sector development. Across the region, inadequate transport infrastructure, limited cold-chain systems, and weak market organization prevent producers from accessing formal and higher-value markets.

The lack of reliable transport services often forces herders to sell milk at the farm gate or through informal channels. In parts of Ethiopia, women frequently sell camel milk along roadsides under conditions that lack hygienic infrastructure and temperature control. Comparable informal marketing systems dominate in Somalia and northern Kenya, where insecurity, poor road networks, and insufficient collection centers restrict commercialization. In Sudan and Djibouti, fragmented market linkages and limited processing facilities similarly constrain formal market expansion.

Limited cold-chain infrastructure, the absence of organized collection centers, and insufficient processing capacity have collectively hindered the establishment of a structured, sustainable camel milk value chain across the Horn of Africa. These structural weaknesses reduce producer bargaining power and limit value addition. Compared with relatively more developed pastoral milk systems in parts of Kenya, other countries in the region exhibit lower levels of market integration and processing capacity. Such regional differences demonstrate the importance of targeted investments in infrastructure, cooperative marketing systems, and regulatory frameworks to strengthen competitiveness and cross-border trade.

To address these constraints, institutional initiatives such as the Ethiopian Meat and Milk Institute (EMMI) and the Pastoral Resilience Improvement through Market Expansion (PRIME) project have provided training and technical support to improve milk handling, marketing, and business skills (Gebremichael et al., 2019). However, similar large-scale and sustained institutional interventions remain limited in Somalia, Sudan, Kenya, and Djibouti, resulting in uneven development of support systems across the region.

Beyond production and marketing, camel milk processing presents additional technological constraints across the Horn of Africa. Camel milk exhibits poor stability under UHT treatment, weak rennet coagulation due to low κ-casein content, extended fermentation time to reach target acidity, and sensitivity to pH changes affecting whey protein stability (Seifu, 2023). These physicochemical limitations complicate industrial-scale processing and product diversification throughout the region, not only in Ethiopia but also in Somalia, Kenya, Sudan, and Djibouti, where processing infrastructure remains limited.

Addressing these production, regulatory, marketing, and technological challenges through coordinated regional policy frameworks, infrastructure investment, and capacity-building will be essential to enhancing value addition, strengthening market integration, and improving the sustainability of the camel dairy sector across the Horn of Africa.

### Opportunities and prospects for camel milk production

The growing demand for camel milk across the Horn of Africa and globally presents significant opportunities for investment, product diversification, and export expansion (Al Abri & Faye, 2019). Within the region, countries such as Kenya, Ethiopia, and Somalia have demonstrated increasing commercialization through peri-urban dairy development, expansion of pasteurized milk markets, and emerging private processing enterprises, indicating tangible pathways toward sector growth. With adequate infrastructure, policy support, and technical training, Ethiopia and neighboring Horn of Africa countries, including Kenya, Somalia, Sudan, and Djibouti, have the potential to transform camel milk into a competitive regional commodity that supports food security, employment, and resilience.

Camel milk has often been perceived as challenging to process and is traditionally consumed mainly as fresh or spontaneously fermented milk (Berhe et al., 2017). However, technological advances and applied research are gradually overcoming processing limitations. Studies demonstrate the feasibility of producing soft cheese, yoghurt, butter, and other value-added products from camel milk under optimized conditions (Ahmed and Kanwal, 2004; Mehaia, 2002; Berhe et al., 2017). In Kenya and Ethiopia, pilot-scale processing facilities and private enterprises have introduced pasteurized milk and yoghurt into urban markets, illustrating practical examples of commercialization beyond traditional consumption systems. These developments support a gradual transition from informal fresh-milk markets toward structured processing systems and scalable enterprises (Sallam, 2020).

Across the Horn of Africa, expanding peri-urban dairies and cross-border milk trade further demonstrate emerging commercialization opportunities. In northern Kenya and eastern Ethiopia, peri-urban aggregation centers link pastoral producers to urban consumers, strengthening supply chain integration. Similarly, informal cross-border trade between Somalia, Ethiopia, and Kenya reflects strong regional demand and market interconnectivity. Although progress is evident, scaling remains uneven due to infrastructural constraints and limited investment.

Camels offer distinct production and marketing advantages over other livestock species (Kena, 2022). In pastoral lowlands, declining cattle productivity under recurrent drought has increased reliance on camels, which maintain milk production during prolonged dry periods. Comparable adaptive shifts have been documented in arid zones of northern Kenya, Somalia, and parts of Sudan, where climate variability reinforces the strategic importance of camels as climate-resilient dairy animals. This resilience underscores camels’ comparative advantage in arid and semi-arid environments, where sustained productivity is critical for long-term sector growth and market stability.

Cultural preferences further strengthen market demand. Sisay and Awoke (2015) report that pastoral communities often prefer camel milk over cow milk, associating it with strength, endurance, and suitability for nomadic lifestyles. Such culturally embedded consumption patterns generate stable local demand and provide a foundation for commercialization. Additionally, growing health awareness and recognition of the nutritional and functional properties of camel milk have expanded its appeal in urban and niche export markets.

To fully capitalize on these opportunities, coordinated improvements in processing technology, cold-chain infrastructure, quality assurance systems, and policy harmonization are required. Streamlining regulatory frameworks across Horn of Africa countries, strengthening regional quality standards, and supporting public–private partnerships would enhance market integration and reduce fragmentation. Targeted investment in research, capacity building, and institutional support will enable the sector to transition from predominantly informal marketing systems toward more competitive regional and global market participation. With sustained technological innovation and policy alignment, the camel milk sector can contribute significantly to food security, employment creation, climate adaptation, and economic empowerment across pastoral communities.

## Conclusion

Camel milk production remains a vital livelihood activity for pastoral and agro-pastoral communities in Ethiopia and across the Horn of Africa, including Somalia, Kenya, Sudan, and Djibouti. It plays a crucial role in sustaining households during prolonged dry seasons when other livestock are less productive. From a compositional perspective, camel milk is characterized by high levels of bioactive proteins, elevated vitamin C and mineral content, a favorable whey-to-casein ratio, and relatively low fat and lactose levels, attributes that enhance its nutritional, functional, and therapeutic value while strengthening its market potential.

Despite these advantages, safety and quality concerns persist due to traditional handling practices, limited cold-chain infrastructure, weak regulatory enforcement, and fragmented quality standards across the region. The camel milk value chain remains largely informal in most Horn of Africa countries, with limited coordination among producers, processors, and distributors, inadequate processing facilities, and restricted access to structured markets. These structural constraints limit commercialization, product diversification, and regional trade integration.

To unlock the sector’s full potential, priority actions should focus on strengthening regional policy harmonization, improving hygiene and quality control systems, investing in processing and cold-chain infrastructure, and promoting technological innovation adapted to camel milk’s unique physicochemical properties. Enhanced cross-border collaboration, research investment, and institutional coordination are essential to transition the sector from predominantly informal systems toward competitive regional and export-oriented markets. With targeted research, technological adaptation, and coordinated institutional support, camel milk can play a transformative role in enhancing food security, climate resilience, employment generation, and inclusive economic development throughout the Horn of Africa.

## Data Availability

No datasets were generated or analysed during the current study.
